# Gel Immersion Endoscopic Submucosal Dissection Using a Scissor‐type Knife for Superficial Non‐ampullary Duodenal Epithelial Tumors

**DOI:** 10.1002/deo2.70157

**Published:** 2025-06-30

**Authors:** Osamu Dohi, Naoto Iwai, Hayato Fukui, Mayuko Seya, Tomoko Ochiai, Junki Yumoto, Hiroki Mukai, Katsuma Yamauchi, Hajime Miyazaki, Takeshi Yasuda, Takuma Yoshida, Tsugitaka Ishida, Hiroaki Kitae, Yukiko Morinaga, Reo Kobayashi, Ryohei Hirose, Ken Inoue, Naohisa Yoshida, Kazuhiko Uchiyama, Tomohisa Takagi, Hideyuki Konishi, Yoshito Itoh

**Affiliations:** ^1^ Molecular Gastroenterology and Hepatology Graduate School of Medical Science Kyoto Prefectural University of Medicine Kyoto Japan; ^2^ Department of Gastroenterology Akashi City Hospital Akashi Japan; ^3^ Department of Gastroenterology Saiseikai Shiga Hospital Ritto Japan; ^4^ Department of Gastroenterology Omihachiman Community Medical Center Omihachiman Japan; ^5^ Department of Surgical Pathology Graduate School of Medical Science Kyoto Prefectural University of Medicine Kyoto Japan; ^6^ Department of Infectious Diseases Graduate School of Medical Science Kyoto, Prefectural University of Medicine Kyoto Japan

**Keywords:** adverse event | endoscopic submucosal dissection | gel immersion | scissor‐type knife | superficial non‐ampullary duodenal epithelial tumor

## Abstract

**Objectives:**

This study aimed to compare the short‐term therapeutic outcomes between conventional endoscopic submucosal dissection (C‐ESD) and gel immersion ESD (GI‐ESD) for superficial non‐ampullary duodenal epithelial tumors (SNADETs).

**Methods:**

A retrospective analysis was conducted on patients with SNADETs who underwent C‐ESD or GI‐ESD between June 2016 and May 2024. To reduce proficiency bias, the first 50 cases per endoscopist were excluded. C‐ESD was performed using a scissor‐type knife under CO_2_ insufflation, while GI‐ESD was performed using the same knife under gel immersion. Primary outcomes included en bloc and R0 resection rates; secondary outcomes were resection time, adverse events, and inflammatory response.

**Results:**

Overall, 51 C‐ESD and 49 GI‐ESD procedures were analyzed. Both groups achieved 100% en bloc resection. R0 resection rates were comparable (C‐ESD: 92.6%, GI‐ESD: 90.2%, *p* = 0.661). Muscle layer exposure was significantly lower in the GI‐ESD group (1.9%) than in the C‐ESD group (16.7%, *p* = 0.032). The mean white blood cell count was also significantly lower in the GI‐ESD group (*p* = 0.038). The incidence of adverse events in the C‐ESD and GI‐ESD groups was 5.6% and 1.9%, respectively (*p* = 0.627). However, no cases of perforation or aspiration were observed in the GI‐ESD group.

**Conclusions:**

GI‐ESD is a safe and effective alternative to conventional ESD for SNADETs, offering comparable resection outcomes and low risk of adverse events with a reduced risk of muscle layer exposure.

## 1 Introduction

Duodenal cancer is extremely rare, with an incidence of only 2.9–4.3 persons per million per year [1, 2, 3]. However, the incidence of superficial non‐ampullary duodenal epithelial tumors (SNADETs) has been increasing in Western and Eastern countries [4, 5]. This may be attributed to advanced endoscopic technologies and increased awareness. Endoscopic resection is an alternative treatment for SNADETs with a low risk of lymph node metastasis. According to the clinical practice guidelines for duodenal cancer, endoscopic submucosal dissection (ESD) is an option for SNADETs, including adenoma, Tis, and T1a cancers > 20 mm [6].

ESD is a reliable treatment for SNADETs as it results in a high rate of en bloc resection with good short‐ and long‐term outcomes while having a low rate of local recurrence [7, 8]. However, due to the anatomical characteristics of the duodenum and poor scope maneuverability, high complication and surgical conversion rates were noted [9, 10]. The development of duodenal ESD was recently reported in several high‐volume Japanese centers, although ESD for SNADETs is only performed by a few expert endoscopists, even in Japan [7, 11, 12]. This suggests that even for endoscopists with extensive experience in non‐duodenal ESD, mastery of duodenal ESD requires at least 50 cases [7, 12].

In recent years, ESD with water pressure method (WPM‐ESD) has been explored as an alternative method to overcome the challenges of duodenal ESD [13]. These techniques with saline immersion can improve endoscopic visualization and submucosal access through mucosal buoyancy and reduced intraluminal pressure. However, they also present limitations such as unstable visual fields due to bile contamination and air bubbles, as well as the risk of fluid‐related adverse events including fecal incontinence and aspiration [14].

Gel immersion endoscopy was developed to secure the visual field using a transparent gel [15]. The advantages of gel immersion ESD (GI‐ESD) include the buoyancy effect of the mucosa due to the gel and low‐pressure conditions due to the lack of air or CO_2_ insufflation [16]. These suggest that GI‐ESD allows us to approach the submucosal layer more easily and to perform the procedure with improved stability and maneuverability than conventional ESD (C‐ESD) [17]. GI‐ESD has been reported in several cases [18, 19]. However, evidence for GI‐ESD for SNADETs is limited. Therefore, our study compared the short‐term outcomes of C‐ESD and GI‐ESD in patients with SNADETs.

## 2 Patients and Methods

### 2.1 Patients

This was a single‐center retrospective study. Consecutive patients with SNADETs who underwent endoscopic resection at Kyoto Prefectural University of Medicine between April 2016 and May 2024 were enrolled. Indications for ESD for SNADETs included preoperative diagnosis of adenomas or adenocarcinomas measuring ≥10 mm, including those with a low risk of lymph node metastasis.

Patients with SNADETs who underwent cold snare polypectomy (CSP), endoscopic mucosal resection (EMR), underwater EMR (UEMR), and laparoscopy and endoscopy cooperative surgery (LECS) as well as those with SNADETs involving the papilla of Vater were excluded. Furthermore, to minimize bias related to proficiency, we excluded the first 50 cases for each endoscopist. All patients provided written informed consent to undergo ESD for SNADETs. Study approval was obtained from the Institutional Review Board of the Kyoto Prefectural University of Medicine (ERB‐C‐1600) according to the ethical standards outlined in the 1964 Declaration of Helsinki and its later amendments.

### 2.2 ESD Procedure

LECS was introduced in our hospital in 2015 and ESD alone was introduced after the stabilization of the ESD procedure during LECS in 2016 [8]. Therefore, ESD has been performed for duodenal lesions measuring ≥10 mm at any location that were initially diagnosed as SNADETs since April 2016. By contrast, SNADETs <10 mm were treated using CSP, EMR, or UEMR during the study period.

**FIGURE 1:**
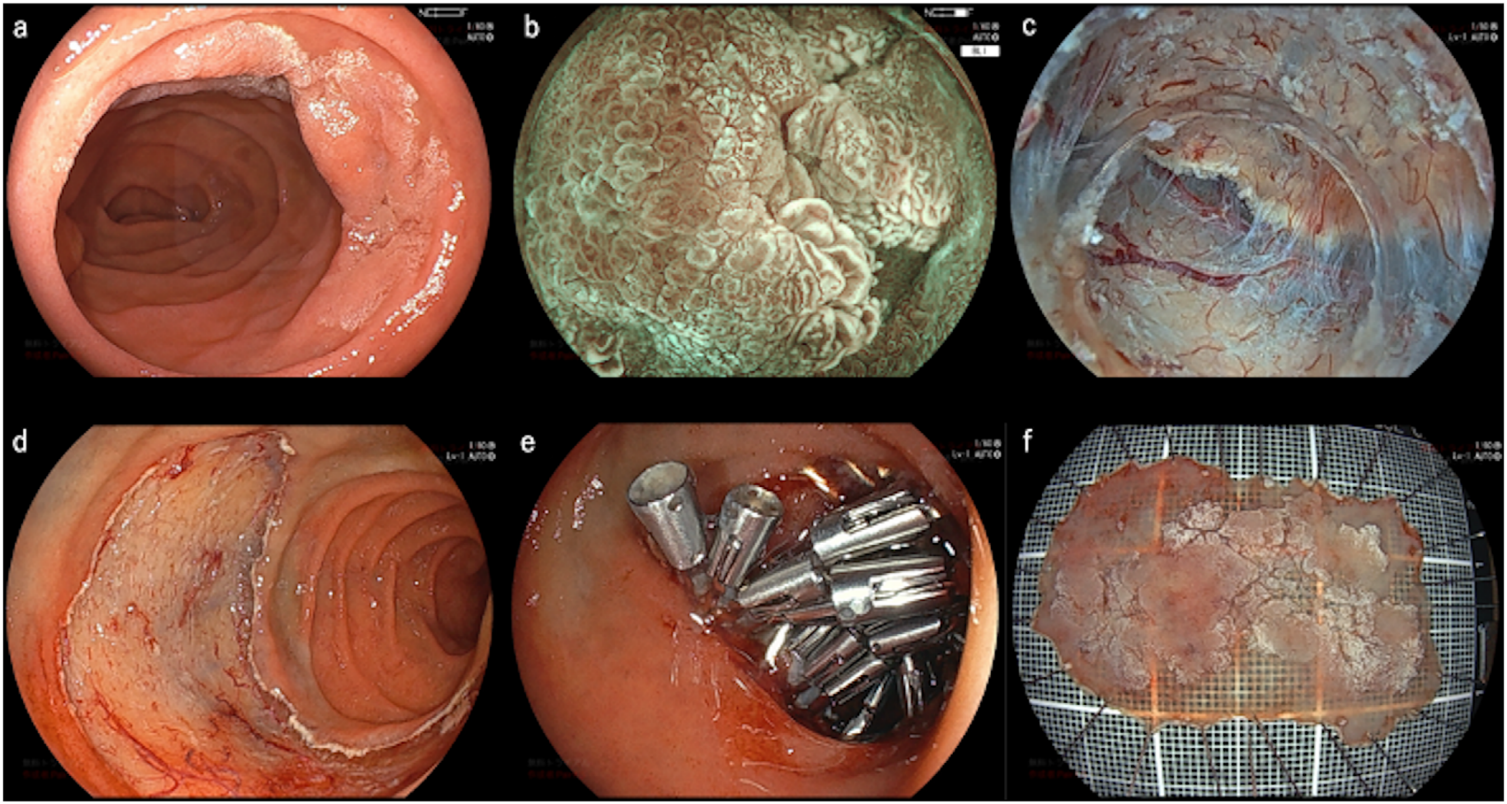
GI‐ESD using a Clutch Cutter for a SNADET: (a) A superficial elevated lesion on the lateral wall of the descending portion. (b) Magnifying blue laser imaging (M‐BLI) shows an irregular microstructure. (c) Submucosal dissection was performed using the pocket‐creation method. (d) ESD was performed without complications. (e) Complete closure was achieved with the underwater clip closure method using 18 reopenable clips. (f) En‐bloc resection was achieved (well‐differentiated adenocarcinoma, 45 mm, pTis, Ly0, V0). SNADET, superficial non‐ampullary duodenal epithelial tumor; ESD, endoscopic submucosal dissection.

**FIGURE 2:**
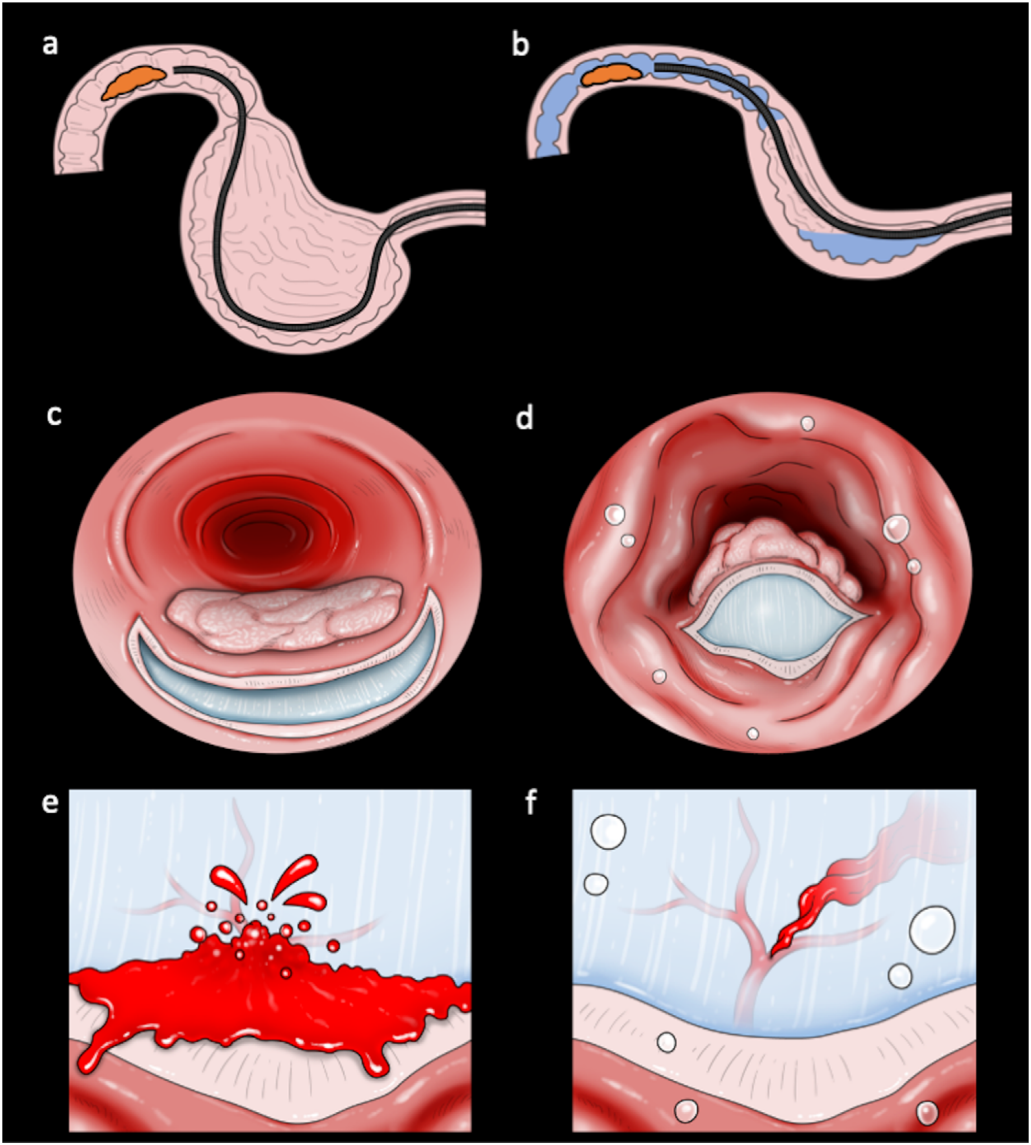
Shema of conventional endoscopic submucosal dissection (C‐ESD) and gel immersion ESD (GI‐ESD): (a) To approach a lesion on the descending portion, the endoscope must be forced into a curved position with CO_2_ insufflation during C‐ESD. (b) GI‐ESD allows the scope to be maintained in a straight position when approaching the lesion due to deflation of the stomach. (c) During C‐ESD, the narrow submucosal space after the mucosal incision makes it difficult for the endoscope to enter the submucosal layer. (d) During GI‐ESD, the submucosal space is widened because the tumor floats into the lumen due to the buoyancy effect. (e) It is difficult to secure a clear visual field when intraoperative bleeding occurs during C‐ESD. (f) Gel immersion maintains a clear visual field because bleeding does not immediately diffuse due to the high viscosity.

Details of C‐ESD performed at our institute have been described previously [11]. During C‐ESD, we used a 3.5‐mm Clutch Cutter as the only ESD device under CO_2_ insufflation from April 2016 to August 2021. From September 2021, GI‐ESD was performed using a 3.5‐mm Clutch Cutter immersed in the gel solution (Viscoclear; Otsuka Pharmaceuticals Factory, Tokushima, Japan) through a BioShield irrigator (U.S. Endoscopy, Mentor, OH, USA) or the accessory channel of the endoscope (EG‐580RD, EG‐580RD7, or EG‐840T; Fujifilm Co., Tokyo, Japan) (Figure 1 and Video S1). To fill the lumen with the gel solution, the gel was injected after deflating the duodenal lumen. When bubbles or bleeding appeared due to mucosal incision and submucosal dissection, the gel was additionally injected to secure the field of view. Figure 2 shows the images and schemas of the different C‐ESD from GI‐ESD conditions.

By the end of 2017, a high‐frequency electrical generator (VIO 300D; Erbe Elektromedizin, Tübingen, Germany) was employed during C‐ESD with the following settings: Endocut I mode, effect 1, duration 4, interval 1; soft coagulation mode, effect 5, 100 W. Since 2018, a high‐frequency electrical generator (VIO3; Erbe Elektromedizin) was employed in both methods with the following settings: Endocut I mode, effect 1, duration 4, interval 1; and soft coagulation mode, effect 5.0.

Prophylactic closure of the mucosal defect was performed to achieve complete closure of each lesion using conventional clip closure, over‐the‐scope clip (OTSC; Ovesco Endoscopy, Tübingen, Germany) closure [11, 20], underwater clip closure method [21], or the reopenable clip‐over‐the‐line method [22]. When closure was incomplete, endoscopic tissue shielding was performed using a polyglycolic acid sheet with fibrin glue [23].

### 2.3 Definitions

**FIGURE 3:**
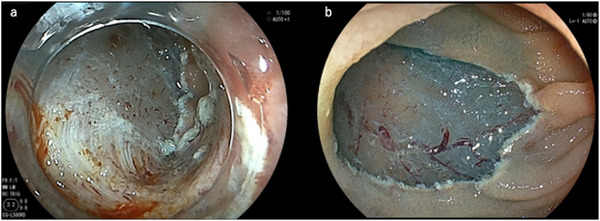
Definition of muscle exposure after endoscopic submucosal dissection (ESD): (a) Muscle layer exposure, positive. The coagulated muscle layer in the mucosal defect is visible. (b) Muscle layer exposure is negative. The muscle layer is not visible in the mucosal defect.

Intraoperative perforation was defined as a full‐thickness defect of the duodenal wall confirmed by direct observation of the peritoneal or retroperitoneal space during endoscopic resection. Muscle exposure was defined as a visible coagulated muscle layer in the mucosal defect (Figure 3). Postoperative bleeding was defined as hematemesis or melena requiring endoscopic hemostasis at the end of the procedure. Postoperative perforation was defined as the detection of a perforation site on endoscopy and/or peritoneal or retroperitoneal emphysema observed on computed tomography upon the manifestation of any symptoms after the procedure.

### 2.4 Participating Endoscopists

All procedures were performed by two skilled endoscopists with experience in performing >200 ESD procedures for upper and lower gastrointestinal tumors and who were accredited by the Japan Gastroenterological Endoscopy Society.

### 2.5 Outcome

The primary outcomes of the study included the en bloc and R0 resection rates for both C‐ESD and GI‐ESD. Secondary outcomes were resection time, prevalence of complete closure, perioperative adverse events, and inflammatory response at postoperative day 1 (white blood cells [WBC] count and C‐reactive protein [CRP] level).

### 2.6 Statistical Analyses

Comparisons between the two groups were performed using the chi‐squared test or Fisher's exact test for nominal variables and the Mann–Whitney U tests or Student's *t*‐tests for continuous variables. Univariate and multivariate analyses using a logistic regression model were performed to evaluate various clinical characteristics and treatment factors associated with muscle layer exposure. Clinically relevant variables with significance in the univariate analysis were also included in the multivariate analysis. Statistical analyses were performed using the Statistical Package for the Social Sciences software (SPSS version 25.0; IBM Corp., Armonk, NY, USA). Statistical significance was set at *p* < 0.05.

## 3 Results

### 3.1 Patient Characteristics

**FIGURE 4:**
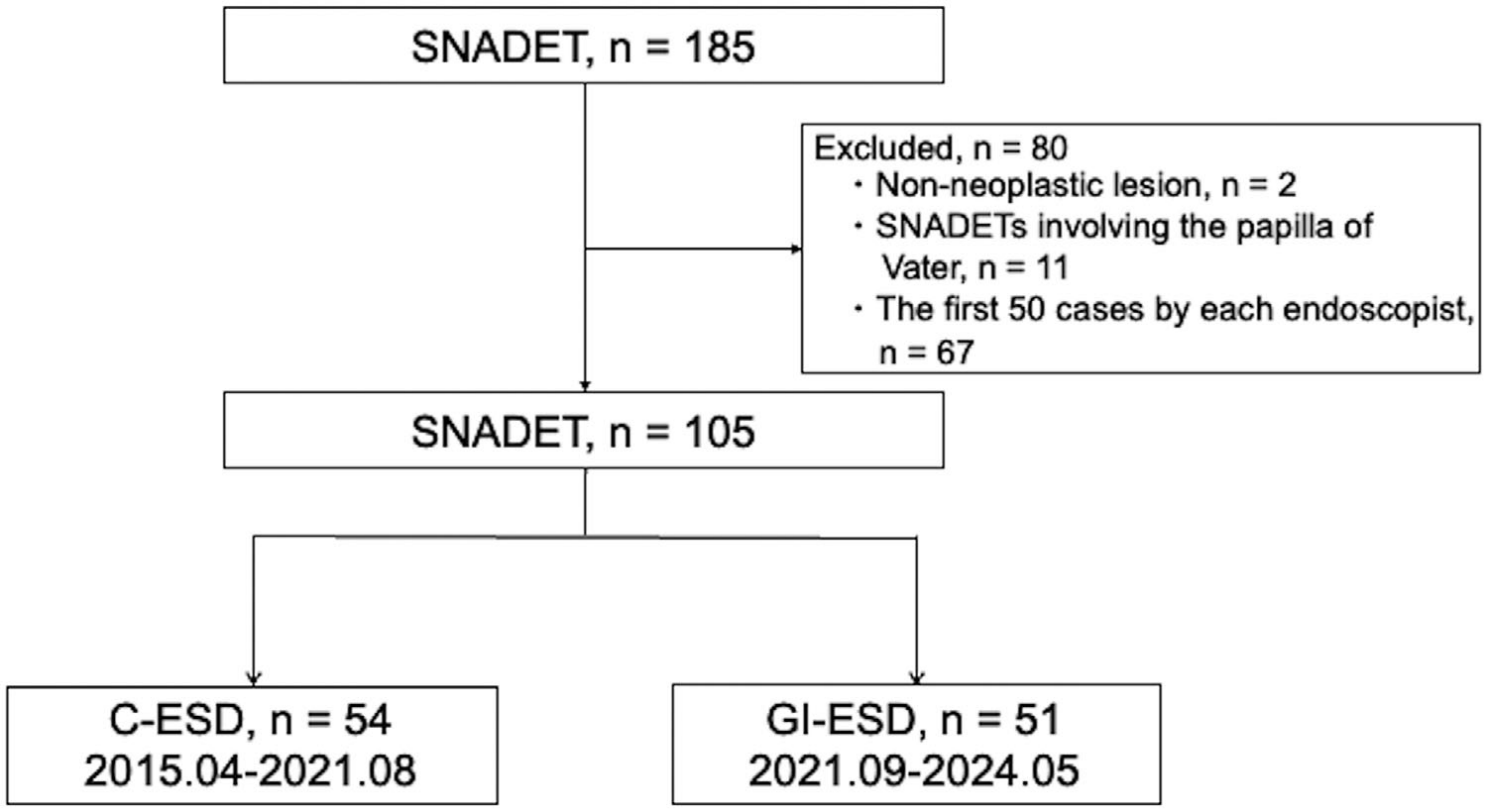
Flowchart of the study population.

Overall, 185 patients who underwent ESD for SNADETs were enrolled in this study. Excluding two patients with non‐neoplastic lesions, 11 patients with SNADETs involving the papilla of Vater, and 67 lesions that were resected in the first 50 cases by each endoscopist, 105 patients remained: 54 and 51 underwent C‐ESD (C‐ESD group) and GI‐ESD (GI‐ESD group), respectively (Figure 4). Patient characteristics and ESD outcomes are shown in Table 1. There were no differences between the two groups in terms of median age, male‐to‐female ratio, tumor location and circumference, median tumor size, preoperative biopsy rate, or initial diagnosis.

**TABLE 1:** Clinicopathological characteristics of patients with superficial non‐ampullary duodenal epithelial tumors (SNADETs).

	C‐ESD,	GI‐ESD,	
Variables	***n* = 54**	***n* = 51**	*p*‐Value
Sex, *n* (%)			0.873
Male	32 (59.3)	31 (60.8)	
Female	22 (40.7)	20 (39.2)	
Age, mean, years, (SD)	65.7 (12.6)	67.2 (11.7)	0.55
Tumor morphology, *n* (%)			0.056
0‐I	6 (11.1)	5 (12.2)	
0‐IIa	38 (70.4)	44 (83.7)	
0‐IIc	10 (18.5)	2 (4.1)	
Location, *n* (%)			0.947
1st portion (bulb, SDA)	14 (25.9)	12 (23.5)	
2nd portion (descending)	27 (50.0)	27 (53.0)	
3rd portion (IDA, transverse)	13 (24.1)	12 (23.5)	
Circumference, *n* (%)			0.852
Anterior	7 (12.9)	4 (7.8)	
Lateral	19 (35.2)	18 (33.3)	
Posterior	11 (20.4)	11 (21.6)	
Medial	17 (31.5)	18 (33.3)	
Tumor size, mean, mm (SD)	25.6 (16.8)	29.7 (15.6)	0.217
Preoperative biopsy, *n* (%)			0.806
Present	33 (61.1)	29 (61.2)	
Absent	21 (38.9)	22 (38.8)	
Initial diagnosis, *n* (%)			0.116
Low‐grade adenoma	16 (29.6)	21 (41.2)	
High‐grade adenoma	1 (1.9)	4 (7.8)	
Adenocarcinoma	37 (68.5)	26 (51.0)	

Abbreviations: IDA, inferior duodenal angle; SD, standard deviation; SDA, superior duodenal angle.

### 3.2 Therapeutic Outcomes

There were no significant differences between the two groups in terms of anesthesia and pathological diagnosis, including histopathology and lymphovascular invasion (Table 2). The en bloc and R0 resection rates were 100% vs. 100% and 92.6% vs. 90.2% in the C‐ESD and GI‐ESD groups, respectively, with no significant difference. There was also no significant difference in the mean resection time between the C‐ESD and GI‐ESD groups (57.9 vs. 55.1 min, respectively). The underwater clipping closure method was significantly more frequently employed in the GI‐ESD group than in the C‐ESD group (90.3% vs. 35.2%, respectively, *p* < 0.001); however, there was no significant difference in the complete closure rate between the two groups (100% vs. 100%, respectively). The rate of muscle layer exposure was significantly higher in the C‐ESD group than in the GI‐ESD group (16.7% vs. 1.9%, respectively, *p* = 0.032). Moreover, the incidence of major adverse events in the C‐ESD and GI‐ESD groups was 5.6% and 1.9%, respectively (*p* = 0.627). Moreover, there was no intraoperative perforation, delayed perforation, or aspiration pneumonia in the GI‐ESD group. For GI‐ESD, gel solution was totally used from 400 to 1800 mL per lesion.

**TABLE 2:** Therapeutic outcomes of endoscopic submucosal dissection (ESD) for superficial non‐ampullary duodenal epithelial tumors (SNADETs).

	C‐ESD,	GI‐ESD,	
Variables	***n* = 54**	***n* = 51**	*p*‐Value
Anesthesia			0.217
General anesthesia	7 (13.0)	3 (5.9)	
Intravenous anesthesia	47 (87.0)	48 (94.1)	
Final pathological diagnosis, *n* (%)			0.165
Adenoma	0 (0)	0 (0)	
Intramucosal cancer	52 (96.3)	51 (100)	
Submucosal cancer	2 (3.7)	0 (0)	
Lymphovascular invasion, *n* (%)	0 (0)	0 (0)	1
Resection time, mean, min (SD)	57.9 (38.5)	55.1 (37.8)	0.721
En bloc resection, *n* (%)	54 (100)	51 (100)	1
R0 resection, *n* (%)	50 (92.6)	46 (90.2)	0.661
Closing method			< 0.001
Conventional clip	13 (24.1)	0 (0)	
Underwater clipping	19 (35.2)	46 (90.3)	
Over‐the‐scope clip	22 (40.7)	4 (7.8)	
ROLM	0 (0)	1 (1.9)	
Complete closure, *n* (%)	54 (100)	51 (100)	1
Muscle layer exposure, *n* (%)	9 (16.7)	1 (1.9)	0.032
Major adverse events, *n* (%)			0.627
Intraoperative perforation	1 (1.9)	0 (0)	
Delayed perforation	1 (1.9)	0 (0)	
Delayed bleeding	1 (1.9)	1 (1.9)	
Aspiration pneumonia	0 (0)	0 (0)	

Abbreviations: CRP, C‐reactive protein; ROLM, reopenable clip over the line method; SD, standard deviation; WBC, white blood cells.

### 3.3 Risk Factors and Inflammatory Response of Muscle Layer Exposure

**FIGURE 5:**
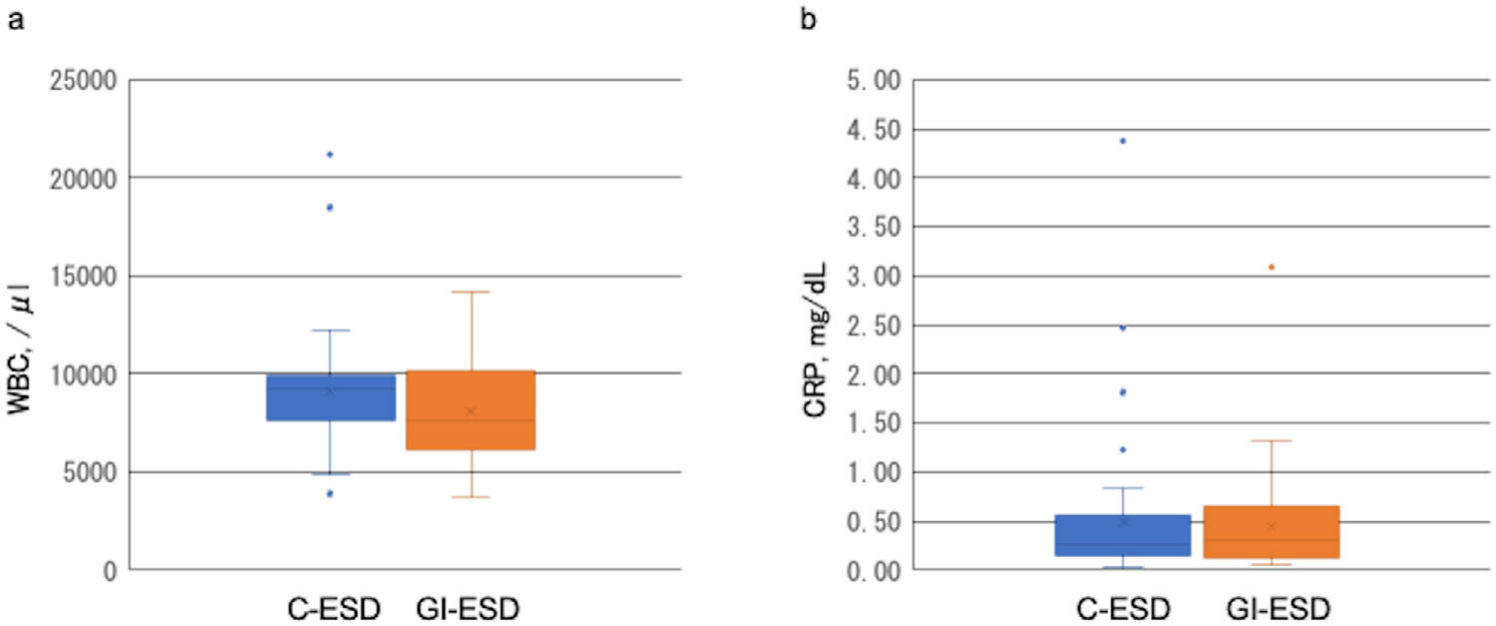
Inflammatory response after C‐ESD and GI‐ESD: (a) white blood cell (WBC) count and (b) C‐reactive protein (CRP) level. C‐ESD, conventional endoscopic submucosal dissection; GI‐ESD, gel immersion endoscopic submucosal dissection.

Univariate logistic analysis revealed that GI‐ESD decreased the risk of muscle layer exposure (odds ratio, 0.100; 95% confidence interval, 0.012–0.821, *p* = 0.032) (Table 3). Moreover, the mean WBC count was significantly different between the C‐ESD and GI‐ESD groups (9135 ± 2955 vs. 8123 ± 2552 µL, respectively, *p* = 0.038). However, the mean CRP level was not significantly different between the two groups (Figure 5).

**TABLE 3:** Logistic regression analyses for risk factors of muscle layer exposure.

Variables	Muscle layer exposure, *n* = 10	No exposure, *n* = 95	Odds ratio	95% CI	*p*‐Value
Tumor morphology					
Elevated	9	84	1.179	0.136–10.214	0.881
Depressed	1	11	Ref		
Location					
Oral side of the papilla	3	47	0.979	0.266–3.604	0.975
Anal side of the papilla	7	48	Ref		
Circumference					
Lateral	6	31	3.097	0.814–11.778	0.097
Others	4	64	Ref		
Tumor size					
≥20 mm	7	60	1.361	0.331–5.605	0.669
<20 mm	3	35	Ref		
Preoperative biopsy					
Present	8	54	3.037	0.612–15.070	0.174
Absent	2	41	Ref		
Anesthesia					
General anesthesia	2	8	2.719	0.492–15.037	0.252
Intravenous anesthesia	8	87	Ref		
ESD method					
GI‐ESD	9	45	0.100	0.012–0.821	0.032
	C‐ESD	1	50	Ref	

Abbreviations: C‐ESD, conventional endoscopic submucosal dissection; CI, confidence interval; ESD, endoscopic submucosal dissection; GI‐ESD, gel immersion endoscopic submucosal dissection.

## 4 Discussion

To the best of our knowledge, this is the first report on the short‐term outcomes of GI‐ESD for SNADETs. GI‐ESD was maintained at adequate en bloc/R0 resection rates but had a significantly lower muscle exposure rate and WBC count than C‐ESD. No periprocedural perforation, such as intraoperative or delayed perforation, occurred in the GI‐ESD cases during the perioperative period. These results suggest that GI‐ESD may reduce the incidence of major adverse events during ESD for SNADETs.

The gel immersion technique offers several advantages that contribute to safer and more effective ESD, particularly in anatomically challenging duodenal locations. Notably, gel immersion ensures a clear visual field, provides mucosal buoyancy, and stabilizes endoscopic maneuverability by maintaining lower intraluminal pressure [16, 17, 18]. In contrast, conventional gas insufflation extends the gastric wall and results in increased intraluminal pressure, making stable endoscope manipulation difficult, especially in the duodenum. These advantages are comparable to those observed in WPM‐ESD [13]. However, saline immersion frequently leads to visual impairment due to the admixture of bile and air bubbles. Gel immersion, by virtue of its viscosity, maintains a stable and clear field of view even in the presence of bile or bleeding, facilitating precise identification of the dissection line and bleeding points. Furthermore, the gel immersion technique requires a relatively small volume of gel to achieve these benefits. While previous reports on WPM‐ESD with saline immersion for SNADETs have indicated increased risks of adverse events—such as fecal incontinence and oral regurgitation—when large volumes of water (median: 2550 mL) and high infusion rates were used [14], our study demonstrated that a gel volume of ≤1800 mL per lesion was sufficient, with no cases of fecal incontinence, regurgitation, or aspiration pneumonia observed. Taken together, these findings suggest that gel immersion is a safer and more efficient alternative to water immersion for duodenal ESD, offering superior visibility and procedural stability with a reduced risk of procedure‐related complications.

We previously performed saline or OS‐1 jelly immersion using a Clutch Cutter during ESD in animal models. However, ESD procedures, including mucosal incision, dissection, and coagulation, could not be performed using a Clutch Cutter because of current dissipation to saline or the OS‐1 jelly, which includes the electrolyte [15]. To overcome this disadvantage, we introduced a dedicated electrolyte‐free GI‐ESD using a Clutch Cutter with no current dissipation. Therefore, gel immersion using Viscoclear was suitable for the Clutch Cutter‐ESD procedure.

A multicenter study showed that the rates of intraoperative and postoperative perforations for duodenal ESD were 9.3% and 2.3%, respectively [7]. In our study, the rate of intraoperative perforation was extremely low for both the C‐ESD and GI‐ESD groups; moreover, GI‐ESD had a lower tendency for postoperative perforation than C‐ESD. Muscle layer injury, including exposure or tears, is a risk factor for post‐ESD electrocoagulation syndrome in colorectal ESD [24]. Moreover, injury to the muscle layer, with muscularis propria exposure and whitish color change, is a risk factor for non‐cardiac chest pain after esophageal ESD [25]. In our study, thermal damage by high‐frequency devices can be transmitted to the muscle layer or deeper layers of the duodenal rumen, leading to a higher degree of inflammatory response that may cause delayed adverse events. Therefore, we should perform duodenal ESD carefully to avoid muscle layer exposure. As shown by our study, GI‐ESD may prevent muscle layer exposure during ESD procedures.

GI‐ESD incurs additional material costs due to the use of dedicated gel products, priced at approximately 2000 Japanese Yen (JPY) per 200 mL bag (approximately USD 15 at current exchange rates). As this was a retrospective study, the precise amount of gel used per procedure could not be determined; however, the total cost of gel per lesion did not exceed 18,000 JPY. This is notably higher than the cost associated with CO₂ insufflation in C‐ESD or saline use in WPM‐ESD. Importantly, minimizing complications such as perforation, delayed bleeding, or aspiration pneumonia can significantly reduce the need for additional interventions, prolongation of hospitalization, and overall healthcare expenditures. Thus, the higher upfront cost of gel may be offset by enhanced procedural stability, improved safety, and potential reductions in downstream medical costs. A prospective cost‐effectiveness analysis is warranted to fully assess the economic impact and clinical value of GI‐ESD in routine practice.

This study had several limitations. First, this was a single‐center, retrospective, non‐randomized, controlled study. Second, the GI‐ESD group had a small sample size. Third, ESD was performed by only an expert endoscopist at a high‐volume center. Fourth, the technical learning curve of C‐ESD and GI‐ESD may be different during this study period regardless of excluding the initial ESD cases. Fifth, differences in the closing method between C‐ESD and GI‐ESD may have resulted in differences in postoperative complications while having comparable complete closure rates.

In conclusion, GI‐ESD for SNADETs is acceptable in terms of the high en bloc and R0 resection rates while having a low incidence of adverse events. GI can reduce the risk of muscle layer exposure compared to CO_2_ insufflation.

## Conflicts of Interest

Osamu Dohi, Naohisa Yoshida, and Tomohisa Takagi received research funds from Fujifilm Co., Ltd. Naohisa Yoshida received lecture fees from Fujifilm Co., Ltd. The other authors declare no conflicts of interest.

## Ethics Statement

**Approval of the research protocol by an Institutional Reviewer Board**: The Ethics Review Committee of the Kyoto Prefectural University of Medicine.

## Consent

All patients provided written informed consent of ESD for SNADETs.

## Clinical Trial Registration

N/A.

## Supporting information




**VIDEO S1** Gel‐immersion endoscopic submucosal dissection was performed using a Clutch Cutter for a superficial non‐ampullary duodenal epithelial tumor measuring 45 mm and located on the lateral wall of the descending portion. The Clutch Cutter‐ESD procedure was performed using the same maneuver: (1) injection of the gel solution after evacuating the air in the duodenal lumen; (2) submucosal injection from the oral side of the lesion; (3) mucosal incision using a Clutch Cutter from the oral side; (4) submucosal dissection using the pocket‐creation method; (5) circumferential mucosal incision; and (6) submucosal dissection for the remaining tissue.

## References

[1] W. Höchter , J. Weingart , H. J. Seib , et al., “Duodenal Polyps. Incidence, Histologic Substrate and Significance,” Deutsche Medizinische Wochenschrift 109 (1984): 1183–1186. 6745123 10.1055/s-2008-1069345

[2] J. M. Jepsen , M. Persson , N. O. Jakobsen , et al., “Prospective Study of Prevalence and Endoscopic and Histopathologic Characteristics of Duodenal Polyps in Patients Submitted to Upper Endoscopy,” Scandinavian Journal of Gastroenterology 29 (1994): 483–487. 8079103 10.3109/00365529409092458

[3] D. Schottenfeld , J. L. Beebe‐Dimmer , and F. D. Vigneau , “The Epidemiology and Pathogenesis of Neoplasia in the Small Intestine,” Annals of Epidemiology 19 (2009): 58–69. 19064190 10.1016/j.annepidem.2008.10.004 PMC3792582

[4] V. H. Coupland , H. M. Kocher , D. P. Berry , et al., “Incidence and Survival for Hepatic, Pancreatic and Biliary Cancers in England Between 1998 and 2007,” Cancer Epidemiology 36 (2012): e207–14. 22534487 10.1016/j.canep.2012.03.010

[5] M. Yoshida , Y. Yabuuchi , N. Kakushima , et al., “The Incidence of Non‐ampullary Duodenal Cancer in Japan: The First Analysis of a National Cancer Registry,” Journal of Gastroenterology and Hepatology 36 (2021): 1216–1221. 33002211 10.1111/jgh.15285

[6] K. Nakagawa , M. Sho , M. Fujishiro , et al., “Clinical Practice Guidelines for Duodenal Cancer 2021,” Journal of Gastroenterology 57 (2022): 927–941. 36260172 10.1007/s00535-022-01919-y PMC9663352

[7] M. Kato , Y. Takeuchi , S. Hoteya , et al., “Outcomes of Endoscopic Resection for Superficial Duodenal Tumors: 10 Years' Experience in 18 Japanese High Volume Centers,” Endoscopy 54 (2022): 663–670. 34496422 10.1055/a-1640-3236

[8] M. Seya , O. Dohi , N. Iwai , et al., “Short‐ and Long‐term Outcomes of Endoscopic Submucosal Dissection and Laparoscopic and Endoscopic Cooperative Surgery for Superficial Non‐ampullary Duodenal Epithelial Tumors,” Surgical Endoscopy 38 (2024): 1784–1790. 38286838 10.1007/s00464-023-10666-x

[9] J. H. Lee , P. Kedia , S. N. Stavropoulos , et al., “AGA Clinical Practice Update on Endoscopic Management of Perforations in Gastrointestinal Tract: Expert Review,” Clinical Gastroenterology and Hepatology 19 (2021): 2252–2261. 34224876 10.1016/j.cgh.2021.06.045

[10] O. Dohi , M. Kato , Y. Takeuchi , et al., “Clinical Course and Management of Adverse Events After Endoscopic Resection of Superficial Duodenal Epithelial Tumors: A Multi‐center Retrospective Study,” Digestive Endoscopy 35 (2023): 879–888. 36945191 10.1111/den.14552

[11] O. Dohi , N. Yoshida , Y. Naito , et al., “Efficacy and Safety of Endoscopic Submucosal Dissection Using a Scissors‐type Knife With Prophylactic Over‐the‐scope Clip Closure for Superficial Non‐ampullary Duodenal Epithelial Tumors,” Digestive Endoscopy 32 (2020): 904–913. 31883154 10.1111/den.13618

[12] Y. Ozeki , K. Hirasawa , A. Sawada , et al., “Learning Curve Analysis for Duodenal Endoscopic Submucosal Dissection: A Single‐operator Experience,” Journal of Gastroenterology and Hepatology 37 (2022): 2131–2137. 36066185 10.1111/jgh.15995

[13] M. Kato , Y. Takatori , M. Sasaki , et al., “Water Pressure Method for Duodenal Endoscopic Submucosal Dissection (With Video),” Gastrointestinal Endoscopy 93 (2021): 942–949. 32853646 10.1016/j.gie.2020.08.018

[14] Y. Takada , T. Hirose , K. Nishida , et al., “Fecal Incontinence and Oral Regurgitation During Duodenal Endoscopic Submucosal Dissection Using the Water Pressure Method,” Digestive Endoscopy 34 (2022): 526–534. 34185924 10.1111/den.14070

[15] T. Yano , A. Ohata , Y. Hiraki , et al., “Development of a Gel Dedicated to Gel Immersion Endoscopy,” Endoscopy International Open 09 (2021): E918–24. 10.1055/a-1396-4236 PMC8159604 34079878

[16] K. Yano , T. Yano , M. Nagayama , et al., “Hemostasis of an Actively Bleeding Lesion at the Ileocecal Valve by Low‐pressure Endoscopy Using the Gel Immersion Technique,” VideoGIE 6 (2021): 184–186. 33898898 10.1016/j.vgie.2020.11.019 PMC8058511

[17] Y. Nakano , T. Tashima , R. Jinushi , et al., “Gel Immersion Endoscopic Submucosal Dissection: Clinical Experience With 13 Cases of Superficial Esophageal Cancer,” Endoscopy International Open 10 (2022): E1302–6. 36118637 10.1055/a-1894-0719 PMC9473821

[18] T. Tashima , K. Miyaguchi , R. Terada , et al., “Gel Immersion Endoscopic Submucosal Dissection Using a Novel Gel Product for a Duodenal Epithelial Tumor,” Endoscopy 54 (2022): E162–3. 33910254 10.1055/a-1443-4796

[19] T. Tashima , T. Ogawa , T. Kawasaki , et al., “Successful Endoscopic Resection by Using Gel Immersion and the Technique of Endoscopic Papillectomy for a Tumor Adjacent to the Papilla of Vater,” VideoGIE 7 (2022): 312–317. 36117941 10.1016/j.vgie.2022.04.002 PMC9479369

[20] T. Tashima , K. Ohata , E. Sakai , et al., “Efficacy of an Over‐the‐scope Clip for Preventing Adverse Events After Duodenal Endoscopic Submucosal Dissection: A Prospective Interventional Study,” Endoscopy 50 (2018): 487–496. 29499578 10.1055/s-0044-102255

[21] T. Ishida , O. Dohi , M. Seya , et al., “Underwater Clip Closure Method for Mucosal Defects After Duodenal Endoscopic Submucosal Dissection (With video),” Digestive Endoscopy 36 (2024): 215–220. 37983598 10.1111/den.14724

[22] T. Nomura , S. Sugimoto , N. Tsuda , et al., “Mucosal Defect Closure After Duodenal Endoscopic Submucosal Dissection Using the Reopenable‐clip Over the Line Method,” JGH Open 5 (2021): 831–833. 34263081 10.1002/jgh3.12577 PMC8264231

[23] H. Doyama , K. Tominaga , N. Yoshida , et al., “Endoscopic Tissue Shielding With Polyglycolic Acid Sheets, Fibrin Glue and Clips to Prevent Delayed Perforation After Duodenal Endoscopic Resection,” Digestive Endoscopy 26 (2014): 41–45. 24750147 10.1111/den.12253

[24] T. Omori , T. Funasaka , N. Horiguchi , et al., “Injury to the Muscle Layer, Increasing the Risk of Post‐colorectal Endoscopic Submucosal Dissection Electrocoagulation Syndrome,” Journal of Gastroenterology and Hepatology 38 (2023): 87–93. 36200387 10.1111/jgh.16021

[25] T. Tahara , T. Shijimaya , S. Nishimon , et al., “Injury to the Muscle Layer and Risk of Non‐cardiac Chest Pain After Endoscopic Submucosal Dissection for Esophageal Cancer,” Journal of Gastrointestinal and Liver Diseases 33 (2024): 25–29. 38386890 10.15403/jgld-5133

