# Supplementary material for: Gel Immersion Endoscopic Submucosal Dissection Using a Scissor‐type Knife for Superficial Non‐ampullary Duodenal Epithelial Tumors
Source: DEN Open. 2025 Jun 30;6(1):e70157. doi: 10.1002/deo2.70157 (PMC12208102; doi:10.1002/deo2.70157)
Supplement: Supplementary file 1 — VIDEO S1 Gel‐immersion endoscopic submucosal dissection was performed using a Clutch Cutter for a superficial non‐ampullary duodenal epithelial tumor measuring 45 mm and located on the lateral wall of the descending portion. The Clutch Cutter‐ESD procedure was performed using the same maneuver: (1) injection of the gel solution after evacuating the air in the duodenal lumen; (2) submucosal injection from the oral side of the lesion; (3) mucosal incision using a Clutch Cutter from the oral side; (4) submucosal dissection using the pocket‐creation method; (5) circumferential mucosal incision; and (6) submucosal dissection for the remaining tissue. [file DEO2-6-e70157-s001.docx]

Video link:

<https://youtu.be/DXqtLpkGick>
